# Outcomes in video laryngoscopy studies from 2007 to 2017: systematic review and analysis of primary and secondary endpoints for a core set of outcomes in video laryngoscopy research

**DOI:** 10.1186/s12871-019-0716-8

**Published:** 2019-04-04

**Authors:** Jochen Hinkelbein, Ivan Iovino, Edoardo De Robertis, Peter Kranke

**Affiliations:** 1 Department of Anaesthesiology and Intensive Care Medicine, University Hospital of Cologne, Kerpener Str. 62, 50937 Köln, Germany; 2 Department of Neurosciences, Reproductive and Odontostomatological Sciences, University of Naples “Federico II”, Via S. Pansini, 5, 80131 Naples, Italy; 3 Department of Anaesthesia and Critical Care, University Hospital of Wuerzburg, Wuerzburg, Germany; 4 Department of Surgical and Biomedical Sciences, University of Perugia, Perugia, Italy

**Keywords:** Airway management, Video laryngoscopy, Primary outcome, Primary endpoint

## Abstract

**Background:**

Airway management is crucial and, probably, even the most important key competence in anaesthesiology, which directly influences patient safety and outcome. However, high-quality research is rarely published and studies usually have different primary or secondary endpoints which impedes clear unbiased comparisons between studies. The aim of the present study was to gather and analyse primary and secondary endpoints in video laryngoscopy studies being published over the last ten years and to create a core set of uniform or homogeneous outcomes (COS).

**Methods:**

Retrospective analysis. Data were identified by using MEDLINE® database and the terms “video laryngoscopy” and “video laryngoscope” limited to the years 2007 to 2017. A total of 3351 studies were identified by the applied search strategy in PubMed. Papers were screened by two anaesthesiologists independently to identify study endpoints. The DELPHI method was used for consensus finding.

**Results:**

In the 372 studies analysed and included, 49 different outcome categories/columns were reported. The items “*time to intubation*” (65.86%), “*laryngeal view grade*” (44.89%), “*successful intubation rate*” (36.56%), “*number of intubation attempts*” (23.39%), “*complications*” (21.24%), and “*successful first-pass intubation rate*” (19.09%) were reported most frequently. A total of 19 specific parameters is recommended.

**Conclusions:**

In recent video laryngoscopy studies, many different and inhomogeneous parameters were used as outcome descriptors/endpoints. Based on these findings, we recommend that 19 specific parameters (e.g., “time to intubation” (inserting the laryngoscope to first ventilation), “laryngeal view grade” (C&L and POGO), “successful intubation rate”, etc.) should be used in coming research to facilitate future comparisons of video laryngoscopy studies.

## Background

Airway management is at least one crucial but probably even the most important key competence in anaesthesiology, which directly influences the safety and outcome of anaesthetised patients [1, 2]. Fortunately, anaesthesia-specific mortality has been significantly decreasing over the last decades and is now estimated to be approximately 1 per 100,000 cases [3–5]. Airway-related problems were reported to cause approximately 40% of anaesthesia-related deaths [6].

Mortality rate is approximately 5.6 per million general anaesthetics or one per 180,000 patients anaesthetised [7]. Taking these numbers into account, it is not surprising that airway management is a major research focus and each year thousands of studies are published analysing many specific problems during airway management [1]. Several specific patient groups have even a higher risk of problems [2].

Endotracheal intubation by using video laryngoscopy has significantly increased over the last decade in both pre- and in-hospital airway management [8]. Today, it is considered standard for difficult airway management and specific emergencies. It is even questioned whether it should be the first choice method.

However, high-quality research is rarely published [1] and studies usually have different primary or secondary endpoints, which impedes high quality comparisons between studies and hampers the possibility to draw meaningful conclusions to significantly and systematically improve safety and quality of clinical care. Different definitions and an inconsistent outcome reporting in studies which investigate comparable clinical problems will, therefore, limit results of research [9–11].

Insufficient attention has been paid on the choice of outcomes used for clinical trials in recent years [12]. To describe and analyse the same intubation performance, some studies use different “time” definitions and intervals (e.g., time to intubation, time to visualize glottis, time to place the endotracheal tube, etc.) and others use anatomical parameters (e.g., Cormack & Lehane grade [13], POGO score [14], etc.). Hence, no standard has been established to facilitate comparisons of results among different studies.

Consensus and consistency when using appropriate outcome measures in clinical trials should enhance the interpretation of research [9]. So far, no conclusive analysis of primary and secondary endpoints being used in studies has been published.

The aim of the present study was to gather and analyse primary and secondary endpoints in video laryngoscopy studies published over the last ten years, i.e., during 2007 to 2017. This data is used to create a basis for development of a core set of outcomes items to be used to facilitate comparisons in future trials. Besides parameters found in published literature, the list would be amended by parameters considered essential in airway management studies.

## Methods

### Systematic PubMed search

Data gathering was performed using MEDLINE® database (http://www.ncbi.nlm.nih.gov/pubmed). To identify relevant literature, the search terms “video laryngoscopy” and “video laryngoscope” were used.

**Fig. 1:**
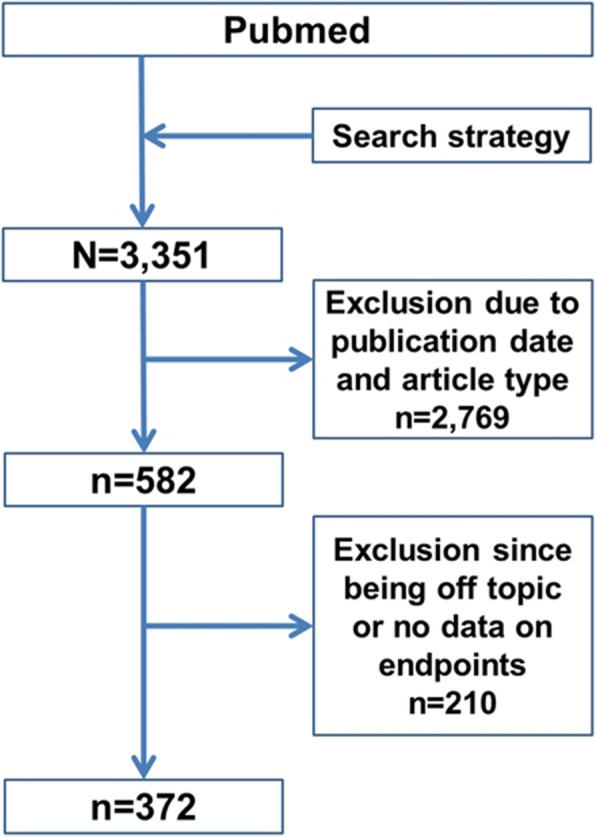
Numbers of studies included and excluded for analysis. A total of *N* = 211 studies provided data on primary and secondary endpoints and were included for analysis

A total of 3351 studies were identified by the applied search strategy in PubMed (Fig. 1). First, a filter restricting the time period of the search (10 years range; going from 22/June/2007 to 22/June/2017) was applied. The PubMed® article categories selected were “Clinical Study”, “Clinical Trial”, “Comparative Study”, “Controlled Clinical Trial”, “Evaluation Studies”, “Multicenter Study”, “Observational Study” and “Randomized Controlled Trial”. The final raw dataset consisted of 582 papers (the number of results is referred to a search made on 11/July/2017).

The complete list of items, including whole article names, authors and PubMed® URLs as well as the table of the results sorted by year was downloaded directly from PubMed® in a CSV format. Manuscripts presenting scientific data on video laryngoscopy as well as outcome parameters were included for analysis. If outcome parameters were not presented, the specific manuscript was excluded for analysis.

### Data analysis

Papers were screened manually by two anaesthesiologists to identify study endpoints. For each study, “primary outcome”/“primary endpoint” or “secondary outcome”/“secondary endpoint” were collected if clearly stated in the abstract and/or in the full text (when available in the University of Naples Federico II or University of Cologne digital libraries).

If a study did not contain parameters described as “primary outcome/endpoint” or “secondary outcome/endpoint”, alternative measurements were included in the analysis. Alternatively, the “aim/goal/objective/target/purpose/null hypothesis” information was used from the abstract. Also, all studies evaluating directly a video laryngoscopy system or evaluating how the effectiveness of these laryngoscopes could be improved by other ancillary devices (e.g., different endotracheal tubes, stylets, gum elastic boogie, etc.) were included.

**Fig. 2:**
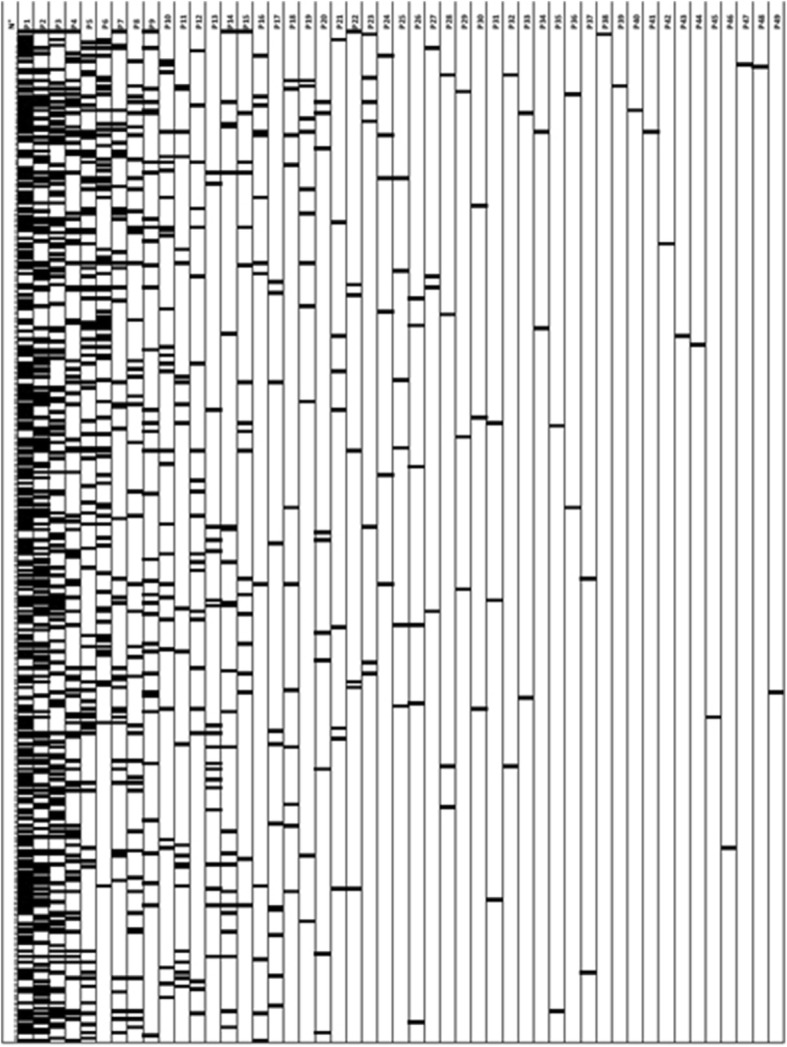
Primary and secondary endpoints of the analysed studies. The parameter with the highest incidence is depicted on the left, with the lowest incidence on the right. This figure shows graphically how often parameters were used in the studies analysed. On the x-axis (columns): from P1 to P49 the different parameters analysed (for their definitions see Table 1). On the y-axis (lines): the specific studies (i.e., *n* = 372). For the percentages of each specific parameters see Table 1

A chess-like table was hence built with the several outcomes as columns and 372 papers included as rows (Fig. 2). Outcomes from different studies, having different names but concerning the same variable were fused in a single column (e.g., “dental compression” and “number of audible dental click sounds”). Outcomes concerning a group of variables, slightly different among the studies but mostly overlapping were fused, as well.

### Data processing

The number of row-column matches for each column/category was reported in a different table and the ratio between it and the total number of included articles was calculated. Derived from the percentages of each parameter, a suggestion was provided of which parameters should be reported in future video laryngoscopy studies to facilitate study comparisons.

### Generation of recommendations

The chosen outcomes outcomes need to be relevant to both health care providers and healthcare users on one hand, and also to those involved in making decisions and choices about health care, on the other hand [12]. However, a lack of attention for using clinical outcomes in studies has led to avoidable losses in both the production and reporting of research. Moreover, the outcomes which have been included in studies have not always been those being most important or relevant for patients [15]. To develop a consensus between the authors concerning use of different parameters, the Delphi system was used [16, 17].

To develop relevant recommendations for video laryngoscopy studies, the four-step Core Outcome Set (COS) process was used. First, the scope was defined, followed by checking if a set exists as a second step. Third, a procedure for the development of the COS was defined and last, it was defined what specific parameters should be measured in future studies [12].

In a COS framework, the method is used for achieving convergence of opinion from experts on the importance of different outcomes in sequential questionnaires (or rounds) sent either by post or electronically [12]. In the present study, all authors (*n* = 4) participated in the process. Three rounds were planned. The answers for each of the outcomes were summarised and fed back anonymously until a consensus was reached with at least 75%. After considering the views of others before re-rating each item, participants were able to change their initial responses based on the feedback from the previous rounds. Direct communication concerning the specific parameters was not possible. Therefore, the feedback provides a mechanism for reconciling different opinions of participants and is essential to achieving a consensus [12]. In terms of the overall validity for the final consensus, this approach has significant advantages as compared to round-table discussions [18].

## Results

### Number of studies

A total of 3351 studies were identified by the applied search strategy in PubMed (Fig. 1). Using the filters for date and article type, a reduction of 2769 papers was obtained. The final raw dataset consisted of 582 papers. During the detailed analysis, 210 papers were excluded because they were considered off topic or belonged to an article type different from the ones chosen during the search filters setting (e.g. meta-analysis, review, etc.). Of the *n* = 210 articles excluded, *n* = 169 (80.48% of 210) were off the topic, *n* = 23 (10.95% of 210) did not evaluate directly a video laryngoscopy system or the effectiveness of the association with ancillary devices, *n* = 13 (6.19% of 210) belonged to a category of articles not included in the search strategy and *n* = 5 (2.38% of 210) did not provide an endpoint.

After exclusion of not relevant papers, *N* = 372 were considered eligible for final analysis (Fig. 1).

### Analysis

The analysis of each item, excluding the off topic articles or the not chosen article types, led to a table consisting of 49 outcome categories depicted horizontally and 372 publications depicted vertically (Fig. 2). The ratio between the number of row-column matches for each outcome category (numerator) and the total number of included articles (denominator) was calculated (the total number of outcome categories was not chosen as denominator since it could be more susceptible to subjective evaluation).

Among the total of 49 collected parameters, the items “*time to intubation*” (65.86%), “*laryngeal view grade*” (44.89%), “*successful intubation rate*” (36.56%), “*number of intubation attempts*” (23.39%), “*complications*” (21.24%), “*successful first-pass intubation rate*” (19.09%) were reported most frequently in the investigated studies (Table 1). Furthermore, these items were grouped in eight parameter categories (Table 1). Besides these six parameters having the highest reporting rate in previous studies and found with the purely computationally approach, additional six parameters were identified in the Delphi round. The Delphi method was used to find a consensus what parameters are of utmost importance. These parameters were “*time to glottis view*”, “*ease of intubation (subjective scoring)*”, “*dental compression AND number of audible dental click sounds*”, “*optimization manoeuvres AND use of airway back-up devices*”, “*haemodynamic parameters*”, and “*lowest arterial oxygen saturation*”. We, therefore, highlighted top twelve of outcomes with a prevalence range from 65.86 to 3.49% (Table 1).

**Table 1:** Frequency of primary and secondary endpoints used in the studies analysed. The “**Top-12**” of the used parameters are indicated in Bold-type

	Outcome Name	Number of Matches	Percent Age	Column Name
Time	**Time to intubation (#1)**	245	65,86%	P1
	**Time to glottic view (#2)**	36	9,68%	P9
	Endotracheal tube insertion time	18	4,84%	P15
	TTSI^a^ on the first attempt	11	2,96%	P18
	Time to ventilation after intubation	7	1,88%	P23
	Total time of chest compression interruption during ETI^b^	2	0,54%	P36
	Time to supraglottic ventilation	1	0,27%	P38
Views	**Laryngeal view grade (CL AND/OR POGO** ^**c**^ **) (#3)**	167	44,89%	P2
Intubation Success	**Successful intubation rate (#4)**	136	36,56%	P3
	**Successful first-pass intubation rate (#5)**	71	19,09%	P6
	**Ease of intubation (subjective scoring)** ^**d**^ **(#6)**	48	12,90%	P7
	Failed intubation	23	6,18%	P11
	Intubation difficulty score (IDS)	22	5,91%	P12
	Factors complicating intubation^e^	11	2,96%	P19
	Proportion of difficult intubation	6	1,61%	P25
	DoubleLumenTube position	4	1,08%	P27
	Successful tracheal intubation rate after failed initial laryngoscopy	4	1,08%	P28
	Intubation success rate in patients with difficult laryngoscopy predictors	2	0,54%	P32
	Reason for intubation failure	2	0,54%	P34
	Likelihood of successful intubation	2	0,54%	P35
	Adequate ETT^f^ position	1	0,27%	P41
	Factors that affect FPS^g^ in trauma patients	1	0,27%	P43
	Proportion of successful to failed intubations	1	0,27%	P45
	Accuracy of correct unilateral placement	1	0,27%	P48
Number of attempts	**Number of intubation attempts (#7)**	87	23,39%	P4
	Number of tube insertions	2	0,54%	P37
Complications	**Complications** ^**h**^ **(#8)**	79	21,24%	P5
	**Dental compression AND number of audible dental click sounds (#9)**	24	6,45%	P14
	Severity of force applied to the upper airway	10	2,69%	P20
	Variables reflecting morbidity^i^	6	1,61%	P24
	Potential laryngeal trauma	2	0,54%	P33
	Gagging severity score at the time of best laryngeal visualization	1	0,27%	P44
Device use & operator variables	**Optimization manoeuvres AND use of airway back-up devices (#10)**	48	12,90%	P8
	Device difficult score	20	5,38%	P13
	Device preference	9	2,42%	P21
	Overall participant satisfaction	7	1,88%	P22
	Ergonomics^j^	3	0,81%	P29
	Postural analysis	3	0,81%	P30
	Learning process	3	0,81%	P31
	Reasons for using methods other than McGrath MAC video laryngoscope	1	0,27%	P39
	Practitioner experience	1	0,27%	P49
Monitoring	**Haemodynamic parameters (#11)**	30	8,06%	P10
	**Lowest arterial oxygen saturation (#12)**	13	3,49%	P16
	Cervical vertebral angle	12	3,23%	P17
	SpO2 immediately after removing the blade from the patient	1	0,27%	P40
	Bispectral index score	1	0,27%	P46
	Intraocular pressure	1	0,27%	P47
Other	Airway grade^k^	6	1,61%	P26
	Intubation conditions^l^	1	0,27%	P42

^a^Time to successful intubation

^b^Endotracheal intubation

^c^Cormack-Lehane score and Percentage Of Glottic Opening

^d^mainly a visual analogue scale score ranging from 1 (extremely easy) to 10 (extremely difficult) with several exceptions (e.g, numerical rating scale 1  =  the easiest, 5  =  the most difficult)

^e^e.g., visualization difficulty related to obscured view from fogging, secretions or blood in the airway; difficulty passing the tracheal tube past the vocal cords; inappropriate endotracheal tube size for the patient; or difficulty controlling the direction of the tracheal tube using the video display

^f^Endotracheal tube

^g^First-pass success

^h^Pre- and post-intubation correlated complictions (e.g., upper airway morbidity, swallowing difficulties or any dental injuries)

^i^e.g., in-hospital mortality, hospital length of stay, duration of mechanical ventilation, duration of ICU stay, ICU mortality, etc

^j^Biomechanical performance of doctors during the ETI (e.g., assessed using surface electromyography and inertial measurement units)

^k^Airway assessment predictors: Mallampati test, mouth opening, thyromental distance, cervical flexion-extension, and neck thickness, snoring, retrognathia, and other types of anomalies also considered as predictors of a difficult airway

^l^Ease of Laryngoscopy, Vocal cords position, Reaction to insertion of the tracheal tube and cuff inflation (Diaphragmatic movement/coughing), direction of the ETT by the forceps and advancement of the ETT by the forceps

Besides the previously reported parameters, seven additional parameters were identified with the Delphi round which should be reported in future airway management studies. Two of them should be reported in any study (patient and manikin study): “exact specifications of the device used” and “exact specifications of the patient group”. For patient studies, the parameters “death”, “ICU admission”, “hospital length of stay”, “dysphagia”, and “reduced quality of life” should be reported.

## Discussion

Over the last ten years, many different and inhomogeneous parameters were used as outcome descriptors/endpoints in video laryngoscopy studies. In order to facilitate literature comparison, taking into account the percentages of items used in previous publications, we suggest that 12 parameters should be used in future video laryngoscopy studies (Table 2). Additionally, the seven patient outcome parameters not covered by the studies before should be reported.

**Table 2:** Suggested minimal endpoints categories for reporting of video laryngoscopy studies. The table consists of 12 previously reported parameters plus seven additional parameters from the Delphi round

Category	Parameter
Time	Time to intubation (taking the laryngoscope to first successful ventilation)
	Time to glottis view
View	Laryngeal view grade (CL and POGO)
Intubation Success	Successful first-pass intubation rate
	Successful intubation rate
	Ease of intubation
Number	Number of intubation attempts
Complications	Any clinically significant complication
	Dental compression AND number of audible dental click sounds
Devices	Optimization manoeuvres AND use of airway back-up devices
Monitoring	Hemodynamic parameters
Patients	Lowest arterial oxygen saturation
Additional parameters (not covered by the studies before)	
Patients outcome	Death
	ICU admission
	hospital length of stay
	dysphagia
	reduced quality of life
Patients	Exact specifications of the patient group
Devices	Exact specifications of the device used

### Video laryngoscopy studies

The use of video laryngoscopes has increased significantly over the last years for many pre-hospital and in-hospital situations [8]. Today, it is considered standard for difficult airway management and emergencies and in some scenarios, it is even questioned whether it should be the method of first choice. Whereas the use of video laryngoscopes was limited to elective intubations several years ago, especially for the anticipated difficult airway, these devices are used today for a broad spectrum of indications such as anticipated difficult airway, teaching and training, or even awake intubation. As airway management is a topic of major research interest and each year thousands of studies that probe all the specific problems of airway management are published, even video laryngoscopy studies seek to compare a variety of devices, arbitrarily chosen, in a variety of settings [1].

In previous studies, a great multiplicity of measured outcomes has been subsequently used to assess the capability of video laryngoscopes to modify the intubation-related variables in comparison to the classic direct laryngoscopy or within the category itself among the different devices [1]. However, none of these outcomes are present in all studies and in some cases, like in the case of the “time to intubation” endpoint, divergence subsists in single definitions, making it difficult to perform any comparison between the outcomes obtained in different articles.

The aim of the study was, therefore, to provide a simple analysis of a part of the scientific literature on the subject so that it would be possible to derive a common basis on which future studies on video laryngoscopy can be built. Standardizing end points will also improve the validity of pooled analysis of clinical trials and assist those wanting to replicate trial results [9]. Besides parameters found in published literature, the list will be amended by parameters considered essential in airway management studies.

## Set of parameters

Nearly no analysed study used the same set of parameters to quantify and qualify performance of intubation with a video laryngoscope. Furthermore, parameters used were often non-specific and not clearly defined, since so far, in this field of research, no consolidated minimal reporting dataset does exist, unlike in other fields of research [19].

**Fig. 3:**
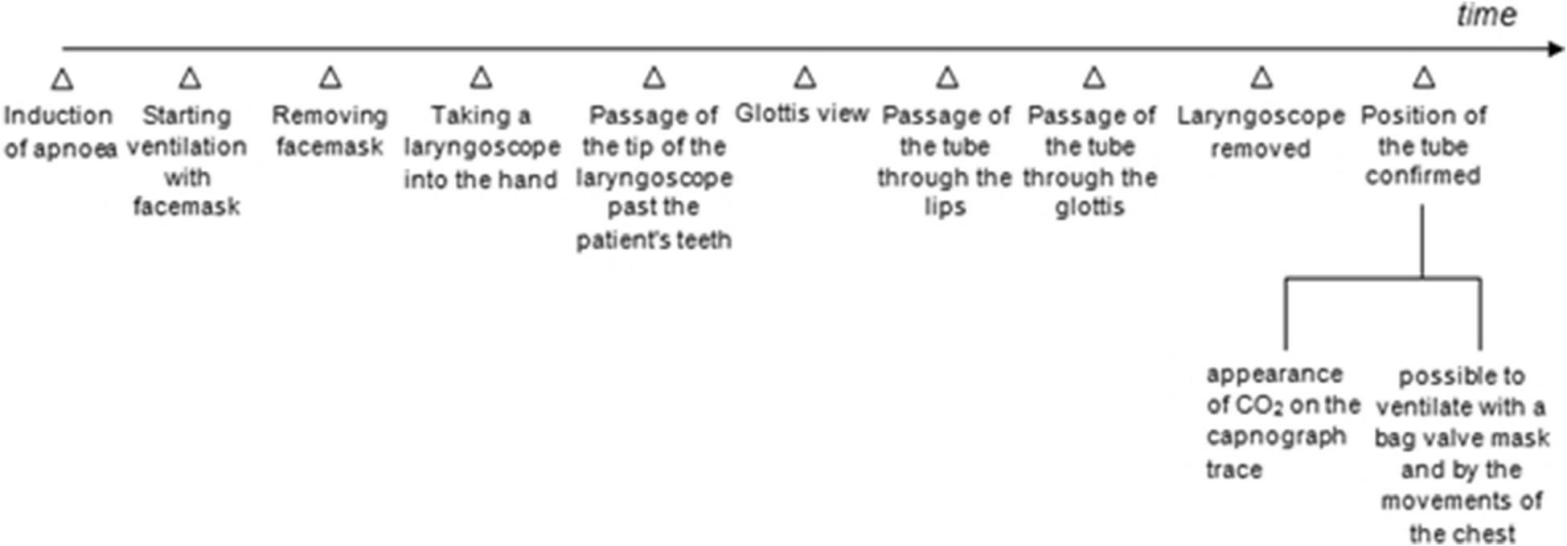
Inhomogeneity among the time points and time frames, related to endotracheal intubation by video laryngoscopy, used in different studies

This problem of definition, even for the meaning of single parameters, is well represented in the main category for prevalence, i.e. “time to intubation”. Three examples could well reflect the high variability in definition since time to intubation is described as (i) the time “from the passage of the tip of the laryngoscope past the patient’s teeth to the appearance of CO_2_ on the capnograph trace” [20], (ii) the moment “from when the facemask was removed from the patient’s face to when end-tidal CO_2_ of at least 20 mm Hg was measured on the end-tidal gas monitor” [21] or when (iii) “the time started to run when a participant took a laryngoscope into his/her hand and stopped when the appropriate position of the tube was confirmed by the fact that it was possible to ventilate with a bag valve mask and by the movements of the chest and the abdomen” (Fig. 3) [22].

## Future aspects

From a practical point of view, several (comparable) parameters and categories should be reported in video laryngoscopy studies (Table 2). This may enhance comparisons of parameters in different studies and facilitate meta-analyses as well as systematic reviews in video laryngoscopy studies. Comparison between studies is made easier, and other investigators will have a stronger foundation on which to design future, definitive trials [9].

## Usefulness of parameters

In the present study, a set of 19 parameters for video laryngoscopy studies is presented. Whereas using many different parameters increases the possibility to compare different studies, it may be cumbersome to record all parameters. Besides this practical point, the feasibility of the different parameters itself varies significantly. Whereas, e.g., “time to intubation”, “number of intubation attempts”, or “successful intubation rate” are quite clear and objective to assess, “ease of intubation” and “hemodynamic parameters monitoring” are far more subjective. Moreover, “laryngeal view grade” or “optimization manoeuvres AND use of airway back-up devices” clearly depends on the skills and expertise as well as anatomical factors of the patient. Therefore, it is essential to keep these limitations also in mind when comparing different studies.

## Limitations

The present study provides an overview on parameters used in previous studies on video laryngoscopy. However, it has also some limitations which should be mentioned. From a total of *n* = 582 articles identified, only *n* = 372 (63.92%) could be included due to the inclusion−/exclusion-criteria mentioned.

Furthermore, not all studies provided information in which model video laryngoscopes were investigated: if in patients, in cadavers, or in manikins. Finally, the definition of video laryngoscopes is quite broad and comparison between the different available models is often impossible.

## Conclusions

Over the years, many different and inhomogeneous parameters were used as outcome endpoints in video laryngoscopy studies.

The final result of parameters offers several recommendations for choosing the endpoints, but the fact remains that these endpoints are still numerous, which reflects the literature on this field although we have demanded a consensus limiting the endpoints to those most relevant and most clinical based from the experts. The example of “Laryngeal view grade” (CL and/or POGO), even if widely cited, does not reflect the difficulty of intubation in video laryngoscopy [23].

The standardization of endpoints for video laryngoscopy studies could lead to improve the effectiveness of literature review and facilitate a more valid comparison between outcomes obtained in future studies.

## Abbreviations

C&L: Cormack&Lehane classification
COS: Core set of uniform or homogeneous outcomes
POGO: Portion of glottis opening score

## References

[1] Hinkelbein J, Greif R, Diemunsch P, Kranke P (2017). Publication and innovation in airway management: quality not quantity!. Eur J Anaesthesiol.

[2] Hinkelbein J (2018). Big data for big patients: gaining insight into risks for tracheal intubation in obese patients. Brit J Anaesth..

[3] Bainbridge D, Martin J, Arango M, Cheng D (2012). Perioperative and anaesthetic-related mortality in developed and developing countries: a systematic review and meta-analysis. Lancet..

[4] Staender S, Mahajan R (2011). Anesthesia and patient safety: have we reached our limits?. Curr Opin Anaesthesiol.

[5] WACKER J, STAENDER S (2014). The role of the anesthesiologist in perioperative patient safety. Curr Opin Anaesthesiol.

[6] Schiff J, Welker A, Fohr B (2014). Major incidents and complications in otherwise healthy patients undergoing elective procedures: results based on 1.37 million anaesthetic procedures. Br J Anaesth.

[7] Cook T, Woodall N, Frerk C, Project FNA (2011). Major complications of airway management in the UK: results of the fourth National Audit Project of the Royal College of Anaesthetists and the difficult airway society. Part 1: anaesthesia. Br J Anaesth.

[8] Hinkelbein J, Cirillo F, Robertis ED, Spelten O (2015). Update on videolaryngoscopy: Most relevant publucations of the last 12 months. Trends Anaesth Crit Care.

[9] Myles P, Grocott M, Boney O, Moonesinghe S, Group C-S (2016). Standardizing end points in perioperative trials: towards a core and extended outcome set. Brit J Anaesth.

[10] Koroshetz W (2015). A core set of trial outcomes for every medical discipline?. Brit Med J.

[11] Ioannidis J (2014). Howto make more published research true. PLoS Med.

[12] Williamson P, Altman D, Bagley H (2017). The COMET handbook: version 1.0. Trials..

[13] Cormack R, Lehane J (1984). Difficult tracheal intubation in obstetrics. Anaesthesia..

[14] Levitan R, Ochroch E, Kush S, Shofer F, Hollander J (1998). Assessment of airway visualization: validation of the percentage of glottic opening (POGO) scale. Acad Emerg Med.

[15] Chalmers I, Glasziou P (2009). Avoidable waste in the production and reporting of research evidence. Lancet..

[16] RANDCORPORATION. Delphi Method. [cited 2016 April]. Available from: http://www.rand.org/topics/delphi-method.html. Accessed 30 May 2017.

[17] Diamond I, Grant R, Feldman B (2014). Defining consensus: a systematic review recommends methodologic criteria for reporting of Delphi studies. J Clin Epidemiol.

[18] Sinha I, Smyth R, Williamson P (2011). Using the Delphi technique to determine which outcomes to measure in clinical trials: recommendations for the future based on a systematic review of existing studies. PLoS Med.

[19] Apfel C, Roewer N, Korttila K (2002). How to study postoperative nausea and vomiting. Acta Anaesthesiol Scand.

[20] Foulds L, Mcguire B, Shippey B (2016). A randomised cross-over trial comparing the McGrath(®) series 5 videolaryngoscope with the Macintosh laryngoscope in patients with cervical spine immobilisation. Anaesthesia..

[21] Turkstra T, Cusano F, Fridfinnson J, Batohi P, Rachinsky M (2016). Early endotracheal tube insertion with the GlideScope: a randomized controlled trial. Anesth Analg.

[22] Szarpak Ł, Czyżewski Ł, Truszewski Z, Kurowski A (2015). Pentax airway scope AWS-S200 video laryngoscope for child tracheal intubation in a manikin study with 3 airway scenarios. Am J Emerg Med.

[23] Gu Y, Robert J, Kovacs G (2016). A deliberately restricted laryngeal view with the GlideScope® video laryngoscope is associated with faster and easier tracheal intubation when compared with a full glottic view: a randomized clinical trial. Can J Anaesth.

